# High-Altitude Illnesses: Physiology, Risk Factors, Prevention, and Treatment

**DOI:** 10.5041/RMMJ.10022

**Published:** 2011-01-31

**Authors:** Andrew T. Taylor

**Affiliations:** Department of Radiology, Emory University School of Medicine, Atlanta, GA, USA

**Keywords:** Acute mountain sickness, high-altitude pulmonary edema, high-altitude cerebral edema, acetazolamide

## Abstract

High-altitude illnesses encompass the pulmonary and cerebral syndromes that occur in non-acclimatized individuals after rapid ascent to high altitude. The most common syndrome is acute mountain sickness (AMS) which usually begins within a few hours of ascent and typically consists of headache variably accompanied by loss of appetite, nausea, vomiting, disturbed sleep, fatigue, and dizziness. With millions of travelers journeying to high altitudes every year and sleeping above 2,500 m, acute mountain sickness is a wide-spread clinical condition. Risk factors include home elevation, maximum altitude, sleeping altitude, rate of ascent, latitude, age, gender, physical condition, intensity of exercise, pre-acclimatization, genetic make-up, and pre-existing diseases. At higher altitudes, sleep disturbances may become more profound, mental performance is impaired, and weight loss may occur. If ascent is rapid, acetazolamide can reduce the risk of developing AMS, although a number of high-altitude travelers taking acetazolamide will still develop symptoms. Ibuprofen can be effective for headache. Symptoms can be rapidly relieved by descent, and descent is mandatory, if at all possible, for the management of the potentially fatal syndromes of high-altitude pulmonary and cerebral edema. The purpose of this review is to combine a discussion of specific risk factors, prevention, and treatment options with a summary of the basic physiologic responses to the hypoxia of altitude to provide a context for managing high-altitude illnesses and advising the non-acclimatized high-altitude traveler.

High-altitude illnesses encompass the pulmonary and cerebral syndromes that occur in non-acclimatized individuals shortly after rapid ascent to high altitude. The most common of these syndromes is acute mountain sickness (AMS) which is described in the editorial, “See Nuptse and Die”, as “vile at best, fatal at worst and an entity to be avoided”.^1^ Nuptse, meaning west peak, rises next to Mount Everest and is commonly viewed from elevations ranging from 3,000–5,000 meters (Figure 1). Excluding Antarctica, only 2.5% of the world’s land mass lies above 3,000 m, yet these heights attract the tourist, hiker, skier, and mountaineer, many of whom dwell near sea-level.^1^ Millions of visitors travel to high altitudes every year, and, with the growth of ecotourism and global adventure travel, ever-increasing numbers of people of all ages are hiking and climbing to very high and even extreme altitudes (Table 1). At 3,000 m, an altitude commonly encountered in ski resorts, the partial pressure of oxygen (PO_2_) is only about 70% of the value at sea-level; at 5,000 m, this value falls to 50% (Table 2). Many high-altitude travelers will be poorly prepared for their trip and naive about the associated risks. This review has two purposes: the first is to highlight the basic physiologic responses to high-altitude hypoxia to provide a context for understanding high-altitude illnesses; the second is to discuss specific risk factors, prevention, and treatment options for acute mountain sickness (AMS) and the potentially fatal syndromes of high altitude pulmonary and cerebral edema so that physicians and health care professionals can appropriately advise travelers ascending to high altitude. The review is organized by specific topics to allow the reader to quickly identify areas of interest.

**Figure 1:**
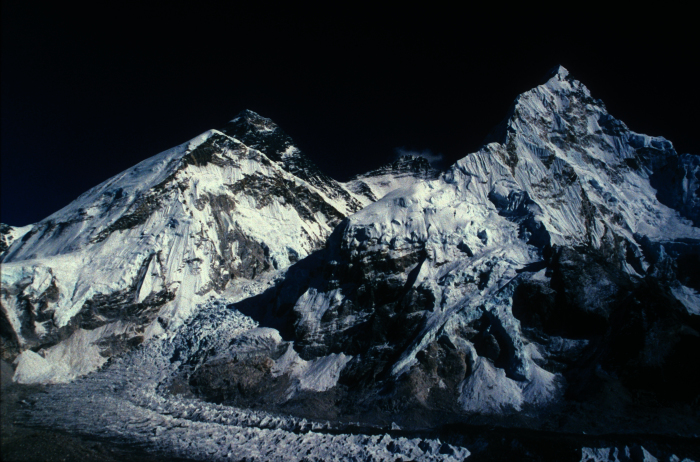
Nuptse on the right, Lhotse in the center, and Mount Everest to the left rear with the Khumbu ice-fall and glacier in the foreground.

**Table 1:** Definitions of high, very high, and extreme altitude.

**Altitude**	**Meters**	**Feet**
High altitude	1,500–3,500	5,000–11,500
Very high altitude	3,500–5,500	11,500–18,000
Extreme altitude	above 5,500	above 18,000

**Table 2:** Changes in barometric pressure and inspired PO_2_ with altitude.*

**Meters**	**Feet**	**Barometric pressure** mmHg	**Inspired PO_2_** (% of sea-level)
0	0	149	100%
1,000	3,281	132	89%
2,000	6,562	117	79%
3,000	9,843	103	69%
4,000	13,123	90	60%
5,000	16,404	78	52%
6,000	19,685	67	45%
7,000	22,966	58	39%
8,000	26,247	51	34%
9,000	29,528	42	28%

* Adapted from West JB. J Appl Physiol 1996;81:1850–4.

## ACUTE MOUNTAIN SICKNESS (AMS)

Acute mountain sickness has been recognized for centuries. As early as two thousand years ago, a Chinese official warned of the dangers of crossing from China into what is now probably Afghanistan. Travelers, he said, would have to cross the “Little Headache Mountain” and the “Great Headache Mountain” where “men’s bodies become feverish, they lose color and are attacked with headache and vomiting”.^2^ Although high altitude is defined as beginning at an elevation of 1,500 m (5,000 feet), symptoms are rarely present at 1,500 m but become increasingly common with rapid ascent to higher elevations. Studies conducted in Nepal, Colorado, Kilimanjaro, and the Alps show a prevalence of AMS ranging from 9% to 58%, with a higher prevalence at higher altitudes (Table 3).^3^–^7^ AMS is typically associated with headache variably accompanied by loss of appetite, disturbed sleep, nausea, fatigue, and dizziness beginning within 12 hours of ascent in two-thirds of susceptible subjects and within 36 hours in the remaining third.^3^ Although more advanced forms of AMS may be accompanied by peripheral edema, periorbital edema, a change in mental status, ataxia, or rales, the initial absence of any definitive signs usually requires clinicians and researchers to rely on subjective symptoms for the diagnosis.

**Table 3:** Prevalence of acute mountain sickness (AMS).

**Author**	**Location**	**Altitude (m)**	**AMS**
Maggiorini 5	Alps	2,85o	9%
		3,050	13%
		3,650	34%
		4,559	53%
Dean 7	Colorado	2,987	42%
Honigman 3	Colorado	2,000–3,000	25%
Vardy 4	Nepal	3,000–4,000	10%
		4,000–4,500	15%
		4,500–5,000	51%
		0ver 5,000	34%
Karinen 6	Kilimanjaro	2,743	9%
		3,760	44%
		4,730	58%

Symptom rating is reasonably reliable for intra-subject evaluation where a person compares his or her current symptoms to a base-line status, but symptom rating becomes much more problematic for inter-subject comparisons since there is no standard of discomfort giving the same score for all subjects. The subjective nature of AMS has resulted in the development of several self-scoring grading systems to determine the presence of AMS and to quantitate its severity.

A very straightforward and common grading system for diagnosing AMS is the Lake Louise self-assessment questionnaire (Table 4), with headache and a score ≥ 3 representing AMS, but other cut-off points and other scoring systems are in common use.^8^–^12^ These scoring systems are not linearly correlated and do not give equivalent results; for this reason, study results are often dependent on the scoring system and cut-off points used to determine the presence or absence of AMS. The literature is further complicated by the fact that many studies are observational investigations, where the many confounding variables (home elevation, rate of ascent, etc.) cannot be taken into account. To avoid the difficulty of controlled and randomized studies in the field, a large number of studies have also been carried out in decompression (hypobaric) chambers.

**Table 4:** Lake Louise self-assessment AMS scoring system.*

1.	**Headache:**	None (0) to incapacitating (3)
2.	**Gastrointestinal symptoms:**	None (0), poor appetite or nausea (1), moderate nausea or vomiting (2), incapacitating severe nausea or vomiting (3)
3.	**Fatigue/weakness:**	None (0) to severe or incapacitating (3)
4.	**Dizziness/lightheadedness:**	None (0) to incapacitating (3)
5.	**Difficulty sleeping (last night):**	None or slept as well as usual (0) to could not sleep at all (3)

* Each symptom is graded on a scale of 0–3; the presence of headache plus a score greater than or equal to 3 is usually considered positive for AMS.^8^

## BAROMETRIC PRESSURE, WATER VAPOR, AND CARBON DIOXIDE

Barometric or atmospheric pressure is usually expressed in mmHg (mercury) although it is occasionally expressed in torr in honor of Evangelista Torricelli (1608–1647) who was the first person to demonstrate that the atmosphere exerts a pressure and can support a column of mercury. One mmHg is essentially equivalent to one torr. At sea-level, the barometric pressure is 760 mmHg. The percentage of oxygen (O**_2_**) in dry air is 20.94%; consequently, the partial pressure of O**_2_** at sea-level is 159 mmHg (0.2094 × 760). When air is inhaled, it is warmed and saturated with water vapor. At 37°C, the saturated vapor pressure in the lungs is 47 mmHg regardless of altitude; because water vapor displaces oxygen and nitrogen, the partial pressure of inspired oxygen is 149 mmHg (0.2094 × (760 – 47)). The partial pressure of water vapor in the lungs at sea-level accounts for only 6% of the total barometric pressure, but water vapor becomes increasingly important at high altitudes. On the summit of Mount Everest, where the barometric pressure is only 250 mmHg, water vapor accounts for nearly 19% of the total barometric pressure, further diminishing the oxygen availability.^13^

At rest, the partial pressure of carbon dioxide (PCO_2_) in the lungs is 40 mmHg, which further displaces oxygen. Although the partial pressure of inspired oxygen at sea-level is 159 mmHg, the combined effects of CO_2_, water vapor, and dead space reduce the partial pressure of oxygen (PO_2_) in the lungs to approximately 100 mmHg. Hyperventilation can reduce the partial pressure of CO_2_ in the lungs below 40 mmHg, thereby allowing the partial pressure of O_2_ to rise. This effect is exaggerated at altitude. On the summit of Mount Everest where the inspired PO_2_ is only 29% of its value at sea-level, alveolar ventilation is increased by a factor of 5. This extreme hyperventilation reduces the alveolar PCO_2_ to 7–8 mmHg, about one-fifth of its normal value. Because of the reduction of PCO_2_, the alveolar PO_2_ can rise and be maintained near 35 mmHg, enough to keep the climber alive.^14^,^15^

## THE HYPOXIC VENTILATORY RESPONSE AND CONTROL OF RESPIRATION

At higher altitudes, the rate and depth of ventilation increase to compensate for the reduced partial pressure of oxygen (PO_2_). This increase in ventilation is termed the hypoxic ventilatory response (HVR) and is partially mediated by the carotid body which is located at the bifurcation of the common carotid artery and is sensitive to dissolved oxygen in the blood. The primary stimulus to breath, however, is not hypoxia; it is hypercapnia, an increase in the level of carbon dioxide in the blood. This stimulus is mediated by potent chemoreceptors located in the medulla. Although the blood–brain barrier separates these medullary chemoreceptors from the arterial blood, the blood–brain barrier is permeable to CO_2_. Increases in the arterial pressure of CO_2_ (PaCO_2_) and hydrogen ion concentration (acidemia) stimulate respiration, and decreases in PaCO_2_ and hydrogen ion concentration (alkalemia) depress respiration.

Through the action of carbonic anhydrase, the CO**_2_** generated in peripheral tissues combines with water to form carbonic acid (H**_2_**CO**_3_**) where it rapidly dissociates into hydrogen and bicarbonate ions as shown below:

$${\text{H}}_{2}\text{O}+{\text{CO}}_{2}\underset{\left(1\right)}{↔}{\text{H}}_{2}{\text{CO}}_{3}\underset{\left(2\right)}{↔}{\text{H}}^{+}+{\text{HCO}}_{3}^{-}$$

The reaction rate of carbonic anhydrase (1) is one of the fastest of all enzymes, and its rate is typically limited by the diffusion rate of the substrates; ionic dissociation (2) is not subject to enzymatic acceleration and is virtually instantaneous. In tissues where there is a high CO**_2_** concentration, the reaction proceeds to the right resulting in increased bicarbonate and hydrogen ion production. The hydrogen ions are buffered by deoxygenated hemoglobin which binds the hydrogen ions and delivers them to the lungs. In the lungs where CO_2_ is being removed, the binding of oxygen by hemoglobin forces the hydrogen ions off the hemoglobin, and the reaction is reversed.

The serum pH is proportional to the bicarbonate/PaCO_2_ ratio. Although the PaCO_2_ depends on the balance between CO_2_ production and CO**_2_** elimination, it is highly dependent on the rate of CO_2_ elimination.^16^

$${\text{PaCO}}_{2}∼\frac{\text{rate}\;\text{of}\;\text{CO2}\;\text{production}}{\text{rate}\;\text{of}\;\text{CO2}\;\text{elimination}}$$

Hyperventilation accelerates CO_2_ elimination and produces a respiratory alkalosis by lowering the PaCO_2_ and raising the pH of the blood. The decrease in PaCO_2_ and the resulting alkalosis combine to act on the medullary chemoreceptor to decrease ventilation. Consequently, the ventilatory response to *hypoxia*, the HVR, becomes especially important in maintaining oxygen saturation, since the normal CO_2_-mediated ventilatory drive is diminished by the hypocapnia. The magnitude and rapidity of onset of the HVR on arrival at altitude varies considerably from individual to individual, and a failure to increase the HVR contributes to hypoxemia and the development of AMS.^17^

## RENAL ADAPTATIONS TO HIGH-ALTITUDE HYPOXIA

As described in the preceding section, the initial response to high-altitude hypoxia is a respiratory alkalosis produced by hyperventilation. Within minutes, the kidneys respond to the alkalosis with an increased excretion of bicarbonate ions; this renal effect can continue for hours or days and functions to correct the alkalosis and return the pH of the serum toward a normal value.

The kidneys also respond to hypoxia by the secretion of erythropoietin. Erythropoietin leads to an increase in red cell mass and the oxygen-carrying capacity of the blood (dissolved oxygen accounts for only about 2% of the oxygen-carrying capacity); however, it takes several days before an increased rate of erythrocyte production can be measured, and the process is not complete for weeks or months.^14^,^18^ For short-term ascents, the erythropoietin-mediated increase in red cell mass is of minor importance, although it is important for extended expeditions. The hematocrit, not the total hemoglobin, is increased during short-term ascents by a reduction in plasma volume caused by a hypoxia-mediated diuresis; the elevation in hematocrit increases the oxygen-carrying capacity per 100 mL of blood.^17^–^20^

## THE HEMOGLOBIN SATURATION CURVE AT ALTITUDE

**Figure 2:**
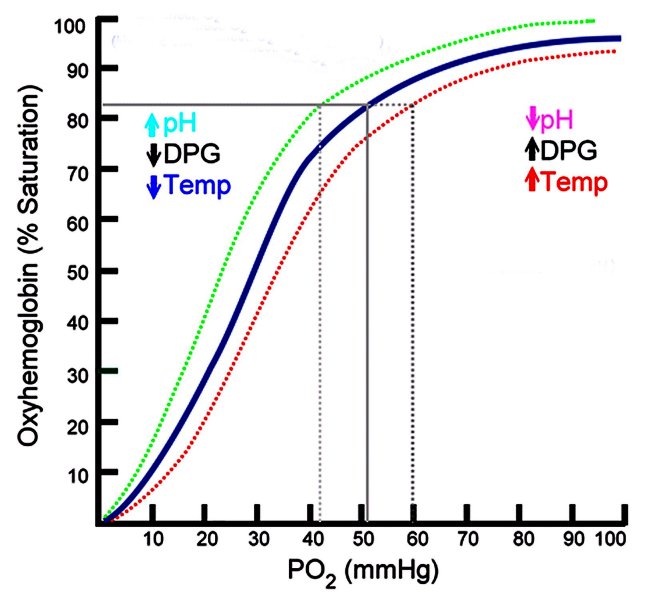
Oxygen-hemoglobin dissociation curve (adapted from reference 21 and used with permission).

When blood is exposed to a high oxygen pressure in the lungs, oxygen rapidly and reversibly combines with hemoglobin to form oxyhemoglobin. At sea-level where the PO_2_ is approximately 100 mmHg, the arterial oxygen saturation of hemoglobin (SaO_2_) is 95%–98%. The oxygen–hemoglobin dissociation curve (Figure 2) shows the changes in hemoglobin saturation as the partial pressure of O_2_ decreases.^21^ Its sigmoidal shape arises from the fact that the hemoglobin molecule contains four heme groups which each react with a molecule of O_2_; oxygenation of the first heme group increases the affinity of O_2_ for the remaining groups. This characteristic shape facilitates oxygen loading in the lungs and oxygen release in the tissues. With increasing altitude, the SaO_2_ is initially well maintained compared to the PO_2_ due to the relatively flat component of the upper portion of the oxygen–hemoglobin dissociation curve. As altitude increases, the steeper section of the oxyhemoglobin dissociation curve assumes a greater importance, resulting in a more rapid decrease in SaO**_2_**. At 8,400 m on Mount Everest where the partial pressure of arterial oxygen (PaO_2_) drops to 25 mmHg, hemoglobin saturation is only 50%.^22^

The increased oxygen demands of actively metabolizing tissues lead to an increased production of CO_2_ and hydrogen ion concentration accompanied by an increase in local temperature and increased levels of 2,3-diphosphoglycerate, all of which shift the oxygen–hemoglobin dissociation curve to the right and facilitate oxygen release in the tissues, while shifts to the left occur under the reverse conditions. At high altitude, the acute respiratory alkalosis arising from hyperventilation causes a leftward shift in the oxygen–hemoglobin dissociation curve, increasing arterial saturation for any given PaO_2_. This leftward shift improves oxygen uptake in the lungs more than it impairs off-loading in the tissues. Under conditions of extreme hypoxia when pulmonary loading is at a premium, the left-shifted increase in hemoglobin oxygen affinity helps maximize the level of tissue oxygenation for a given difference in oxygen tension between the sites of oxygen loading in the pulmonary capillaries and sites of oxygen unloading in the tissue capillaries.^23^

## AMS: CLINICAL FEATURES

The hypoxia of high altitude can lead to sleep disturbances, impaired mental performance, weight loss, and reduced exercise capacity.

## SLEEP

Humans rapidly ascending from sea-level to sleep at altitudes above 2,500 m often experience disturbances in sleep quantity and quality caused by a combination of low arterial oxygen levels and periodic breathing. Periodic breathing, oscillations in respiratory frequency and/or tidal volume, is a well documented phenomenon in normal healthy adults.^24^ Following rapid ascent to high altitude, periodic breathing during sleep is almost universal and contributes to the disturbing dreams, frequent arousals, awakenings, and subjective sense of poor-quality sleep often experienced at altitude.^25^,^26^ The underlying pattern of periodic breathing is exacerbated by hypoxia and amplified by an increased hypoxic ventilatory response. The resulting hyperventilation leads to a hypocapnic alkalosis which can depress ventilation even to the point of apnea. Hypoventilation leads to hypoxia and a further reduction in oxygen saturation which, in turn, stimulates hyperventilation and generates a self-sustaining cycle.^26^ Via its effect on the carotid body, acetazolamide leads to a significant reduction in periodic breathing, improves arterial saturation during sleep at high altitude, and helps to prevent or diminish the symptoms of AMS.^26^ Because of the risk of respiratory depression, sedative hypnotic drugs should be avoided.

## MENTAL PERFORMANCE AND CEREBRAL ATROPHY

The brain normally accounts for 20% of total oxygen consumption. Under the high-altitude conditions of moderate to severe hypoxia, mental performance is impaired.^14^ Impairment in codification and short-term memory is especially noticeable above 6,000 m, and alterations in accuracy and motor speed occur at lower altitudes.^27^ Of greater concern are studies that indicate both amateur and professional climbers ascending to very high and extreme altitudes are at risk for subcortical lesions and cortical atrophy.^28^,^29^

## WEIGHT LOSS AT ALTITUDE

Altitude exposure may lead to considerable weight loss, which appears to be a function of both absolute altitude and the duration of exposure. Physical activity, nausea due to AMS, and lack of palatable food all contribute to weight loss at altitude, and this weight loss can be further exacerbated by gastro-enteritis, upper respiratory infections, and low temperatures. Initial weight losses of approximately 3% occur at elevations below 4,000 m, and weight losses up to 15% may occur during extended stays from 5,000 to 8,000 m.^30^ The initial weight loss likely reflects a diuresis and loss of water. Beyond this initial diuresis, weight loss appears to be preventable by maintaining physical activity and an adequate dietary intake; unfortunately, some trekking companies skimp on the quality and variety of food and contribute to weight loss by failing to provide an adequate diet. Above 5,000 m weight loss is probably unavoidable and is mainly a result of muscle fiber atrophy independent of activity level, possibly related to the direct effects of hypoxia on protein metabolism.^30^,^31^

## PHYSICAL CONDITION AND EXERCISE

Exercise capacity diminishes with altitude. The alveolar partial pressure of oxygen is slightly higher than the partial pressure of oxygen in the arterial blood, and this alveolar–arterial pressure difference widens progressively during exercise in conjunction with an increased cardiac output, shortened capillary transit time, and greater venous oxygen desideration. At sea-level, this exercise-induced pressure differential is accompanied by a ventilatory response that rises out of proportion to increasing oxygen demands; this heightened ventilatory response is usually sufficient to maintain the arterial PO_2_ and prevent the development of hypoxemia.^32^ Under the hypoxic conditions of high altitude, however, the ventilatory response is no longer sufficient to prevent arterial oxygen desaturation with exercise; and even mild arterial desaturation (< 94% SaO_2_) is associated with a significant reduction in maximum oxygen consumption and endurance performance.^33^ Maximum oxygen consumption is reduced to about 85% of its value at sea-level at 3,000 m, and it falls to 60% at 5,000 m.^14^

When combined with rapid ascent, strenuous exercise and over-exertion are risk factors for AMS. In a controlled study of subjects experiencing a simulated altitude gain of 3,000 m in a decompression chamber, exercise significantly reduced arterial saturation (SaO_2_) and increased the AMS symptom scores.^34^ The effect of physical conditioning in preventing AMS is more difficult to evaluate since those in good physical condition are apt to engage in more strenuous exercise and undertake more rapid ascent, both risk factors for AMS. Data suggest, however, that subjects in excellent physical condition probably have a risk of AMS similar to that in less highly trained individuals.^3^,^35^,^36^

## AMS: RISK FACTORS

AMS is associated with a number of potential risk factors including home elevation, maximum sleeping altitude, rate of ascent, latitude, age, gender, physical condition, intensity of exercise, hemoglobin saturation, pre-acclimatization, prior experience at altitude, genetic make-up, and pre-existing diseases.

## HOME ELEVATION AND MAXIMUM SLEEPING ALTITUDE

Travelers ascending from sea-level are at higher risk for AMS than those living at higher elevations. This difference is illustrated by a study at a Colorado ski resort showing that the risk of developing AMS was 27% for residents arriving from sea-level compared to 8.4% for those residing above 1,000 m.^3^ The risk of AMS increases with sleeping altitude; among mountaineers staying at huts in the Swiss Alps, the prevalence of AMS ranged from 9% at 2,850 m to 53% at 4,559 m (Table 2).^5^ These results are comparable to the prevalence of AMS among trekkers staying at tea houses in Nepal which ranged from 10% at 3,000–4,000 m to 51% at 4,500–5,000 m (Table 2).^4^ Interestingly, in this study, the prevalence of AMS decreased from 51% at 4,500–5,000 m to 34% above 5,000 m (Table 2) and was likely due to self-selection or prior experience at altitude among those ascending above 5,000 m.

## RATE OF ASCENT AND KILIMANJARO

A rapid rate of ascent is an important contributor to the development of AMS.^3^ Trekkers in the Everest region of Nepal appear to have a slower rate of ascent and a lower prevalence of AMS compared to those climbing Kilimanjaro where the rate of ascent is more rapid.^4^,^6^,^37^,^38^ In climbers ascending to very high altitudes, differences of a few days in acclimatization can have a significant impact on the prevalence of AMS, symptom severity, and mountaineering success.^36^

At 5,895 m, Kilimanjaro is the world’s highest free-standing mountain measured from base to summit. It is popular, easily accessible, and its location near the equator offers the option of combining a summit attempt with a safari to neighboring game preserves. Every year 20,000 climbers try to reach the summit.^6^ The standard routes to the summit, with the possible exception of the Western Breech which requires some scrambling, are not technical and can potentially be hiked by anyone in good physical condition. In spite of the non-technical nature of the climb, there have been numerous fatalities on this mountain.^6^ To cut costs and compete effectively, trekking companies often schedule relatively rapid climbs leaving limited time for acclimatization. Of particular concern is the observation that some hikers continue to ascend in spite of developing life-threatening signs of high-altitude pulmonary or cerebral edema.^6^ Although not always practical, current recommendations are to limit the increase in sleeping altitude to 600 m in a 24-hour period once above 2,500 m and to add an extra day of acclimatization for every 600–1,200 m gain in elevation.

## LATITUDE

Latitude affects oxygen availability, hemoglobin saturation, and the risk of developing AMS. Due to its rotation, the Earth bulges at the equator; consequently, both barometric pressure and PO_2_ are higher at the equator than at the poles. On the 6,194 m summit of Denali in central Alaska, the barometric pressure is equivalent to barometric pressure on the summit of a *6,900-m* peak in the Himalayas.^39^ Because of this effect, at an equivalent elevation climbers will be less hypoxic on Kilimanjaro (3°S) or even Everest (23°N) than on Denali (63°N). If Everest had been situated at the same latitude as Denali, it could not have been climbed without supplemental oxygen.

## GENDER AND AGE

Men and women appear to be equally at risk for AMS,^4^,^5^,^39^ although some observational studies suggest a slightly higher risk for women.^3^ Older individuals do not appear to have an increased risk of AMS;^4^,^36^ in fact, one study suggests that younger individuals may be at higher risk. Eighteen-to-nineteen-year-olds had a 45% incidence of AMS at Colorado ski resorts compared to only 16% for those between 60 and 87 years of age.^3^ This study was uncontrolled, and the results are probably affected by a greater exercise intensity in the younger age group. There are no controlled trials of AMS in children, but the attack rate appears similar to that in adults.^40^

## INTENSITY OF EXERCISE

As described above, the alveolar–arterial pressure difference widens progressively with increasing exercise, leading to reduced hemoglobin saturation at altitude with an increase in the risk and severity of AMS.^32^,^34^ To decrease the risk of AMS, strenuous exercise and over-exertion should be avoided immediately after rapid ascent to high altitude.

## ARTERIAL OXYHEMOGLOBIN SATURATION

**Figure 3:**
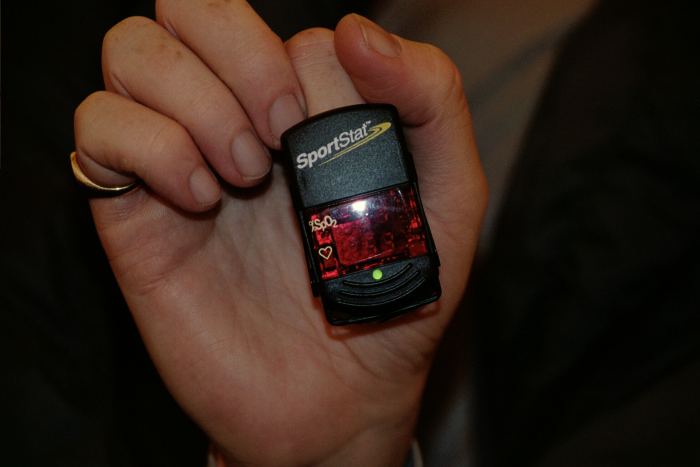
Pulse oximeter.

Early hypoxemia, a decrease in the SaO**_2_** greater than that expected for a given altitude, is a risk factor for developing AMS.^41^–^43^ Early hypoxemia appears to be the result of a diffusion impairment or venous admixture and can be monitored with a pulse oximeter (Figure 3).^41^–^43^ Individuals with early hypoxemia should be advised to avoid strenuous exercise and, if continuing to ascend, to ascend slowly. Pulse oximeters are relatively inexpensive and are commonly carried by trekking companies to monitor SaO**_2_** in individuals with worsening symptoms of AMS; however, if they are to be used at very high or extreme altitudes, it is important to check the calibration. SaO**_2_** measurements below 83% may not have the same degree of accuracy and precision as measurements with higher saturations.^44^

Pulse oximeters have a pair of small diodes that emit light of different wavelengths through a translucent part of the patient’s body such as the finger-tip or ear-lobe; based on differences in absorption of the two wavelengths, the instrument can distinguish between deoxyhemoglobin and oxyhemoglobin. To function properly, the pulse oximeter must detect a pulse since it is calibrated to detect the pulsatile expansion and contraction of the arterial blood vessels with the heart-beat. Inaccurate readings may occur in subjects with frost-bite, cold digits, or hypovolemia.

## PRIOR AMS AND PREVIOUS EXPOSURE TO ALTITUDE

A prior history of AMS is an important predictor for developing AMS on subsequent exposures to comparable altitudes.^45^ Conversely, a history of recent or extreme altitude exposure is associated with a lower risk of AMS (6,962 m).^45^,^46^ Self-selection is likely an important factor; those who tolerate and enjoy the high mountains without developing AMS are more likely to repeat the experience.

## GENETIC ADAPTATIONS

Humans have lived and worked at high altitudes for thousands of years. Perhaps the best known high-altitude populations are the Sherpas and Tibetans in the Himalaya and the Quecha and Ayamara in the Andes. Hemoglobin concentration is higher in the Andean populations than in Himalayan highlanders, whereas Himalayans respond to their hypoxic environment with a higher ventilatory response.^47^ These differences are likely to have a genetic component, although no specific genetic differences have yet been identified.

Many cellular functions such as protein synthesis are down-regulated by hypoxia, but select subsets are up-regulated. Prominent among the up-regulated subsets is the family of genes governed by hypoxia-inducible factor 1.^48^ Hypoxia-inducible factor 1 functions as a global regulator of oxygen homeostasis facilitating both O_2_ delivery and adaptation to O_2_ deprivation. The first-discovered example of hypoxia-dependent gene expression was erythropoietin which leads to an increased hematocrit and O_2_-carrying capacity. Another genetic factor which may contribute to high-altitude performance is a polymorphism in the angiotensin-converting enzyme gene that appears to be more prevalent in elite mountaineers and in endurance athletes than in the general population.^49^ Individuals differ widely in their susceptibility to high-altitude disorders; some suffer the life-threatening complications of high-altitude cerebral or pulmonary edema at altitudes as low as 3,000 m, whereas others can climb to 8,000 m without supplemental oxygen. Genetic influences remain an active area of investigation.^50^

## PRE-EXISTING DISEASES

Recreational travelers, hikers, and skiers with underlying cardiac or pulmonary diseases often seek advice regarding high-altitude travel. Asymptomatic patients with coronary disease generally do well, although it is probably prudent to avoid highly strenuous exercise; patients with heart failure should avoid the hypoxia of high altitude.^51^ Severe anemia and sickle cell disease are also contra-indications to high-altitude travel.^51^ The advice for patients with lung disease depends on the underlying disease, its severity, and the anticipated altitude and activity level; specific recommendations are contained in an extensive review of the subject.^52^

## HIGH-ALTITUDE CEREBRAL EDEMA

High-altitude cerebral edema (HACE) is likely a continuum of AMS. AMS is generally self-limiting, whereas HACE can be fatal. Individuals with high Lake Louise scores should be carefully monitored for the signs of ataxia, confusion, and hallucinations which may mark the onset of HACE. HACE is a clinical diagnosis and consists of ataxia and altered consciousness in someone with AMS or high-altitude pulmonary edema. Individuals with AMS should not ascend until symptoms have resolved; if symptoms fail to resolve, they should descend. Individuals with HACE should descend immediately if at all possible and should never descend unaccompanied.

The exact processes leading to high-altitude cerebral edema are unknown although the edema is probably extracellular, due to blood–brain barrier leakage (vasogenic edema), rather than intracellular, due to cellular swelling (cytotoxic edema).^53^ Vasogenic edema preferentially spreads along white matter tracts, whereas cytotoxic edema affects both gray and white matter. MRI studies of patients with HACE showed that the majority had intense T2 signal in white matter areas, particularly the splenium of the corpus callosum, but no gray matter abnormalities.^53^ The predilection for the splenium and corpus callosum is puzzling. Possibly the splenium has more easily perturbed cellular fluid mechanics than surrounding tissues. Splenium MRI abnormalities are not limited to patients with HACE and occur in settings that include alcohol use, infections, hypoglycemia, and electrolyte abnormalities;^54^ in these cases, abnormalities in the splenium were also associated with confusion and ataxia, and this set of symptoms may be characteristic for edema involving the splenium. The cause of death in HACE is brain herniation.

**Figure 4:**
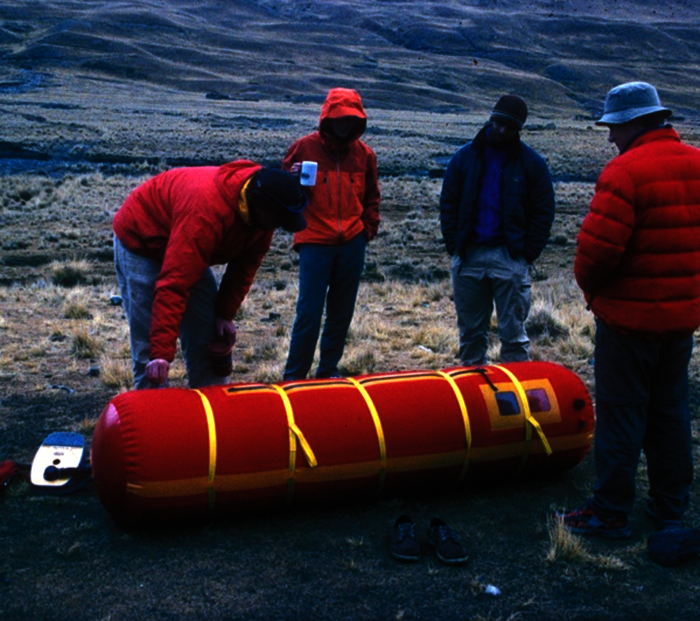
Portable hyperbaric chamber.

Dexamethasone (see below) can be used to treat AMS and HACE, but, unlike acetazolamide, dexamethasone does not facilitate acclimatization and may give a false sense of security. It is an excellent rescue drug to assist in descent.^55^,^56^ If descent is not possible, both oxygen and portable inflatable hyperbaric chambers (Figure 4) improve oxygen saturation and can be effective treatments for subjects with HACE or high-altitude pulmonary edema.^57^,^58^

Inflatable hyperbaric chambers are often carried by trekking companies taking clients to altitude; the bags weigh about 6.5 kg and, when expanded, are cylindrical in shape and large enough to accommodate a person (Figure 4). By inflating the bag with a foot pump, the effective altitude can be decreased as much as 1,500 meters (5,000 feet). The foot pump has to be used continuously while the person is in the bag to supply fresh oxygen and to flush out carbon dioxide.

## HIGH-ALTITUDE PULMONARY EDEMA

High-altitude pulmonary edema (HAPE) is a potentially fatal consequence of rapid ascent to high altitude. Early diagnosis may be difficult since many of the early symptoms (shortness of breath, tachypnea, tachycardia, reduced arterial saturation, fatigue, and cough) are often present in unaffected climbers at higher altitudes, particularly in cold, dry, or dusty environments. Distinguishing features of high-altitude pulmonary edema include incapacitating fatigue, dyspnea with minimal effort that advances to dyspnea at rest, orthopnea, and a dry non-productive cough progressing to a productive cough with pink frothy sputum due to hemoptysis. Fever may also accompany HAPE, and its presence does not imply infection; prompt administration of antibiotics is not required unless other symptoms or a chest radiograph indicate pneumonia.^59^

The onset of HAPE is usually delayed and typically occurs 2–4 days after arrival at altitude; it is not uniformly preceded by AMS.^14^ HAPE is most common at altitudes greater than 3,000 m,^52^ but HAPE can and does occur at lower altitudes. Over a 7-year period, 47 cases of HAPE were reported at a single Colorado ski resort with an elevation of 2,500 m.^60^

The pathogenesis of high-altitude pulmonary edema is still a subject for investigation; however, it is probably triggered by an increase in pulmonary artery pressure due to the normal pulmonary vasoconstriction induced by hypoxia. Patients with HAPE have an enhanced pulmonary reactivity to hypoxia, an exaggerated increase in pulmonary artery pressures, and are improved by pharmacological interventions that decrease pulmonary artery pressure.^61^–^63^ In a subset of individuals, moderate to intense exercise may play a contributory role since exercise independently leads to an increase in pulmonary artery pressures and this effect may be additive to the increased pressures resulting from hypoxia.

Compelling evidence indicates that HAPE is a hydrostatic-induced permeability leak with mild alveolar hemorrhage.^62^,^64^,^65^ Two explanations have been suggested. The first is that that hypoxic pulmonary vasoconstriction is not homogeneous; consequently, pulmonary capillaries supplied by dilated arterioles are exposed to high pressures which cause damage to the capillary walls (stress failure) and leads to a leak of high-protein edema fluid with erythrocytes.^4^ The second explanation hypothesizes an increase in pulmonary capillary pressures due to hypoxic pulmonary venous constriction.^62^,^65^ Regardless of the mechanisms, successful prophylaxis and treatment of high-altitude pulmonary edema using nifedipine, a pulmonary vasodilator, indicates that pulmonary hypertension is crucial for the development of high-altitude pulmonary edema.^63^,^66^

There are no randomized controlled trials evaluating treatment strategies. Oxygen, rest, and descent are commonly agreed upon.^59^,^66^ When patients fail to respond to conservative measures or develop HAPE in remote settings, nifedipine is recommended, 10 mg orally initially and then 30 mg of the extended release formulation orally every 12–24 hours.^66^ Phosphodiesterase inhibitors such as tadalafil have been shown to prevent HAPE in susceptible individuals^67^ and may also be effective in patient management. Some physicians are now employing combination therapy with nifedipine and phosphodiesterase inhibitors,^68^ although these are off-label uses. If descent is not possible, use of a portable hyperbaric chamber is recommended.

## AMS: PREVENTION AND TREATMENT

Drugs used in the prevention and management of AMS include acetazolamide, dexamethasone, phosphodiesterase inhibitors, and analgesics. Strategies to prevent AMS include preacclimatization, copious water consumption, and a high-carbohydrate diet.

## ACETAZOLAMIDE

Acetazolamide is a potent carbonic anhydrase inhibitor; its efficacy in preventing and ameliorating AMS has been well demonstrated although there is still debate regarding the optimal dose.^69^–^71^ A recent double-blind, randomized, placebo-controlled study in the Everest region of Nepal showed that 125 mg twice a day was just as effective in preventing AMS as 375 mg twice a day.^69^ In this study, the incidence of AMS among subjects taking acetazolamide averaged about 22% compared to 51% for those taking a placebo. Acetazolamide is not a panacea; a substantial percentage of subjects taking acetazolamide still develop AMS. In fact, on Kilimanjaro, where the rate of ascent tends to be faster than in Nepal, the incidence of AMS in those taking acetazolamide (250 mg twice a day) was 55% versus 84% for a comparison/placebo group.^72^ Although the precise dose and recommended duration of treatment have never been established,^56^ a reasonable approach for prevention is 125 mg twice a day beginning 1 day prior to ascent and continuing for 2 days after reaching maximal altitude or until descent is initiated; if ascent is rapid, 250 mg twice a day may be more efficacious but carries a greater risk of side-effects. In children, the recommended dose of acetazolamide is 2.5 mg/kg orally given every 12 hours with a maximum dose of 250 mg;^73^ treatment for 48 hours is usually sufficient for resolution of symptoms.^40^

The actual mechanisms by which acetazolamide increases minute ventilation, leads to improvements in arterial blood gases, and reduces the symptoms of AMS remain poorly understood.^71^ The efficacy of acetazolamide has been attributed to inhibition of carbonic anhydrase in the kidneys resulting in bicarbonaturia and metabolic acidosis, which offsets the respiratory-induced alkalosis and allows chemoreceptors to respond more fully to hypoxia stimuli at altitude. Other mechanisms, however, are likely involved: the bicarbonaturia ultimately lowers the cerebral spinal fluid (CSF) bicarbonate concentration, thereby lowering the CSF pH and stimulating ventilation.^71^ Membrane-bound carbonic anhydrase isoenzymes are present on the luminal side of almost all capillary beds including the brain and can be inhibited by low doses of acetazolamide leading to a local tissue retention of CO_2_ in the order of 1–2 mmHg.^71^,^74^ This slight increase in partial pressure of CO_2_ in the brain may stimulate profound changes in ventilation given the high CO_2_ ventilatory responsiveness of central chemoreceptors.^74^ In fact, inhibition of red blood cell and vascular endothelial carbonic anhydrase has been shown to cause an almost immediate retention of CO_2_ in all tissues as the normal mechanisms for exchange and transport are attenuated. The resulting tissue acidosis is postulated to be an important stimulus to the hyperventilation associated with carbonic anhydrase inhibition.^71^,^74^ In addition to improvements in ventilation from tissue acidosis, other operative mechanisms likely include improvements in sleep quality from carotid body carbonic anhydrase inhibition and the effects of diuresis.^71^

Acetazolamide is a sulfonamide drug; patients with an allergic reaction to sulfonamide antibiotics are more likely to have a subsequent allergic reaction to a non-antibiotic sulfonamide drug, but this association appears to be due to a predisposition to allergic reactions rather than to a specific cross-reactivity with sulfonamide-based antibiotics.^75^ Nevertheless, the general recommendation is that patients with known allergies to sulfa drugs should avoid acetazolamide.^56^ The most common side-effects of acetazolamide are peripheral and circumoral paresthesias, but loss of appetite and nausea have been reported. The effect of carbonic anhydrase inhibition in the mouth can also affect the taste of carbonated beverages. Higher doses (250 mg twice or three times a day) are associated with greater side-effects. Finally, the safety of acetazolamide in pregnancy has not been established, and it should be used in pregnancy only if the benefits clearly outweigh the risks.^66^

## DEXAMETHASONE

Dexamethasone is probably less effective than acetazolamide in preventing AMS,^70^ but it is effective as an emergency treatment of AMS in a dosage of 4–10 mg initially, followed by 4 mg every 6 hours.^55^,^56^,^76^,^77^ Dexamethasone reduces AMS symptomatology but does not improve objective physiologic abnormalities related to exposure to high altitudes; a subject with severe AMS may have a dramatic response in symptomatology after treatment with dexamethasone but still show cerebral edema on a CT scan.^77^ At present, dexamethasone is recommended only when descent is impossible or to facilitate co-operation in evacuation efforts.^76^,^77^

## PHOSPHODIESTERASE INHIBITORS

Decreased nitric oxide synthesis may be a contributory factor in HAPE. Nitric oxide, a vasodilator produced in the pulmonary vascular endothelium, has a short half-life as a result of local phosphodiesterase (PDE) activity; consequently, PDE inhibitors enhance the effect of nitric oxide. The 5-PDE inhibitor sildenafil (Viagra) diminishes the pulmonary hypertension induced by acute exposure to hypobaric hypoxia at rest and after exercise,^78^ protects against the development of altitude-induced pulmonary hypertension, and improves gas exchange, limiting the altitude-induced hypoxemia and decrease in exercise performance.^79^ Tadalafil has been shown to prevent HAPE in susceptible individuals,^67^ and this class of drugs shows promise in the management of patients with HAPE.

## ACETAMINOPHEN AND IBUPROFEN

Acetaminophen and non-steroidal anti-inflammatory drugs such as ibuprofen and aspirin are often effective in relieving the headache associated with AMS.^80^,^81^

## HYDRATION

Avoiding dehydration is important, especially since considerable moisture can be lost through respiration at high altitude. Although hypo-hydration degrades aerobic performance at altitude, it does not appear to increase the prevalence or severity of AMS.^82^ Nevertheless, a belief has developed that hypo-hydration increases the risk of AMS and that excessive hydration can prevent or treat the disorder.^83^ Some trek leaders even urge clients to consume excess quantities of water to avoid or ameliorate AMS, but there is no scientific basis for this advice.^66^,^84^ The belief may have originated from observations on the Jungfraujoch (3,471 m) where it was noted that new arrivals passing the greatest quantity of urine tolerated altitude better than those passing the least amount of urine.^83^ This observation may have led to the assumption that consuming large quantities of water would lead to a diuresis and prevent AMS. The early diuresis that occurs at altitude, however, is a response to hypoxia not excess fluid consumption; the development of AMS is associated with a rise in the plasma concentrations of antidiuretic hormone and fluid retention.^19^

## PRE-ACCLIMATIZATION AND ALTITUDE SIMULATION

Pre-acclimatization, spending time at altitude prior to undertaking a higher ascent, reduces the likelihood of developing AMS.^46^ Living at high elevation and training at low elevation improves performance in athletes of all abilities; the primary mechanism is an increase in erythropoietin which leads directly to an increase in red cell mass. The increase in red cell mass allows greater oxygen delivery to the tissues, an increase in maximum oxygen consumption, and an improvement in exercise capacity.^85^,^86^ Pre-acclimatization is usually impractical for the high-altitude traveler or recreational climber, and the “live high, train low” approach is not an option for most athletes. Intermittent hypoxic training has been introduced using normobaric or hypobaric hypoxia in an attempt to reproduce some of the key features of altitude acclimatization and enhance performance.^85^,^87^,^88^ Hypoxia at rest has the primary goal of stimulating acclimatization, while hypoxia during exercise has the goal of enhancing performance.

The simplest intermittent hypoxic training strategy is breathing air with a reduced partial pressure of oxygen under resting conditions; this strategy is straightforward, but unresolved variables are the optimum number of sessions, optimum length of each session, and timing of the sessions prior to ascent. At present, no set of resting, normobaric, hypoxic training parameters have been defined that will reproducibly reduce the likelihood of AMS. A much more sophisticated approach is the use of an altitude simulation system which can safely reduce the oxygen content in a room or tent. This system creates a hypoxic environment that is portable, ideally suited for a “living high, training low” environment and is now used in Olympic training centers around the world.^86^ Red cell transfusions as well as exogenous erythropoietin have been used to increase red cell mass, but neither approach is legal in athletic competition.

## CARBOHYDRATES

Ingestion of pure carbohydrates 40 min prior to acute hypoxic exposure has been shown to improve hemoglobin saturation by as much as 4%; the effect, however, wears off by 150 min, and any advantage of carbohydrate consumption in improving oxygenation is only applicable during the period the carbohydrates are being digested.^89^ This effect depends on the respiratory quotient (RQ) which represents the ratio of carbon dioxide excreted to the amount of oxygen utilized; the value of this ratio depends on the carbon content of food and is typically around 0.85, but it ranges from 0.7 (pure fat) to 1.0 (pure carbohydrates). As shown in the following equation, metabolism of carbohydrates produces a higher PAO_2_ than the metabolism of fat:

$${\text{PAO}}_{2}={\text{PiO}}_{2}-{\text{PaCO}}_{2}/\text{RQ}$$

where PAO_2_ is the partial pressure of oxygen in the alveoli, PiO_2_ is the partial pressure of inspired oxygen, and PaCO_2_ is the partial pressure of carbon dioxide. A higher PAO_2_ will result in a higher hemoglobin oxygen saturation. Effectively, the metabolism of carbohydrates produces a larger quantity of CO_2_ than the metabolism of proteins or lipids;^90^ the increased CO_2_ production provides an added stimulus to the respiratory centers.

## SUMMARY

The typical symptoms of AMS include headache, loss of appetite, disturbed sleep, nausea, fatigue, and dizziness, beginning shortly after rapid ascent to high altitude. The hypoxia of high altitude can lead to sleep disturbances, impaired mental performance, weight loss, and reduced exercise capacity. Factors impacting the risk of AMS include home elevation, maximum altitude, sleeping altitude, rate of ascent, latitude, intensity of exercise, pre-acclimatization, prior experience at altitude, and genetic make-up. Symptoms can usually be relieved by rest and by delaying further ascent until symptoms have resolved; if symptoms are severe, they can be rapidly relieved by descent to a lower elevation. Acetazolamide in doses of 125 mg twice a day reduces the incidence and severity of AMS in areas of relatively slow ascent such as the Everest region of Nepal; under these conditions, higher doses do not appear to be more effective but may be advantageous during the more rapid ascent that occurs on mountains such as Kilimanjaro. AMS may progress to high-altitude cerebral edema (HACE), and high-altitude pulmonary edema (HAPE) may occur in the absence of AMS. Both of these conditions are medical emergencies; if possible, initial management should include descent, supplemental oxygen, and, in the case of HACE, dexamethasone. Nifedipine and phosphodiesterase may be effective in the management of HAPE. A person suspected of either of these conditions should never descend alone. Portable hyperbaric chambers should be considered if descent is not an option.

## Abbreviations:

AMS: acute mountain sickness;
CSF: cerebral spinal fluid;
CT: computed tomography;
H^+^: hydrogen ion;
H_2_CO_3_: carbonic acid;
HACE: high-altitude cerebral edema;
HAPE: high-altitude pulmonary edema;
HCO_3_^−^: bicarbonate;
Hg: mercury;
HVR: hypoxic ventilatory response;
m: meters;
mL: milliliters;
mm: millimeters;
MRI: magnetic resonance imaging;
O_2_: oxygen;
PaCO_2_: partial pressure of arterial carbon dioxide;
PAO_2_: partial pressure of oxygen in the alveoli;
PCO_2_: partial pressure of carbon dioxide;
PDE: phosphodiesterase;
PiO_2_: partial pressure of inspired oxygen;
PO_2_: partial pressure of oxygen;
RQ: respiratory quotient;
SaO_2_: arterial oxygen saturation of hemoglobin.
